# Supplementary material for: A scalable molecule-based magnetic thin film for spin-thermoelectric energy conversion
Source: Nat Commun. 2021 Feb 16;12:1057. doi: 10.1038/s41467-021-21058-x (PMC7887260; doi:10.1038/s41467-021-21058-x)
Supplement: Supplementary file 1 — Supplementary Information [file 41467_2021_21058_MOESM1_ESM.pdf]

Supplementary Information for

A scalable molecule-based magnetic thin film for spin-thermoelectric energy conversion

Oh *et al.*

Supplementary Information for

A scalable molecule-based magnetic thin film for spin-thermoelectric energy conversion

Inseon Oh[1], Jungmin Park[2], Daeseong Choe[1], Junhyeon Jo[1], Hyeonjung Jeong[1], Mi-Jin Jin[1,3], Younghun Jo[2], Joonki Suh[1], Byoung-Chul Min[4], and Jung-Woo Yoo[1*]

[1]Department of Materials Science and Engineering, Ulsan National Institute of Science and Technology, Ulsan, 44919, Korea.

[2]Center for Scientific Instrumentation, Division of Scientific Instrumentation & Management, Korea Basic Science Institute, Daejeon, 34133, Korea.

[3]Soft Chemical Materials Research Center for Organic-Inorganic Multi-Dimensional Structures, Dankook University, Yongin, 16890, Korea

[4]Center for Spintronics, Korea Institute of Science and Technology, Seoul, 02792, Korea.

Correspondence and requests for materials should be addressed to J.-W. Y. (email: jwyoo@unist.ac.kr)

Supplementary Note 1. Materials preparation and characterization

ECD deposition of Cr-PBA

For the deposition of $Cr^{II}[Cr^{III}(CN)_6]$ Prussian blue analogue (Cr-PBA) films, we employed an electrochemical deposition (ECD) method, which can be easily adopted for scalable production of thin films. In order to build a spin thermoelectric (STE) bilayer device, a magnetic insulator needs to be grown on a metallic thin film carrying a high spin Hall angle for the effective spin-charge conversion from the magnon-delivered spin angular moments. However, heavy metals such as Pt, Pd are not appropriate for the working electrode due to hydrogen generation by electrolysis of water at the reduction voltage of Cr^{3+} ($E = -0.88$ V vs Ag/AgCl reference electrode). A Cr metal does not display hydrogen generation during ECD, and it has spin Hall angle comparable to those of heavy metals such as Pt, W, and Pd. Thus, we adapted a Cr thin film (10 nm) as a working electrode, which was deposited on SiO_2 (300 nm)/*p*-Si (500μm) substrate by thermal evaporation at high vacuum (~ 7×10^{-7} Torr). The aqueous solution mixed with 5 mM $K_3Cr(CN)_6$ and 7.5 mM $CrCl_3 \cdot H_2O$ was stirred for 30 min at room temperature in order to sufficiently dissolve the reagents in water (HPLC grade). The Cr-PBA film was grown at a fixed potential ($E = -0.88$ V vs Ag/AgCl reference electrode) through the reaction between $[Cr(CN)_6]^{3-}$ and labile Cr^{2+} in the aqueous solution of $K_3Cr(CN)_6$ and $CrCl_3 \cdot H_2O$. Here, the labile Cr^{2+} was generated at the surface of the Cr electrode by the electrochemical reduction of Cr^{3+}, which is ionized from $CrCl_3 \cdot H_2O$. Typical cyclic voltammetry curves are displayed in Fig S1a. Fig. S1b exhibits recorded static current generated during the deposition of Cr-PBA. After the deposition, samples were rinsed with water and dried with N_2 gas. The film thickness was determined by using a surface profiler (P-6 stylus profiler, KLA Tencor). For the sample of 600 sec ECD deposition, average thickness was ~ 1.4 μm. Surface morphology was probed by atomic force microscopy (AFM). The obtained rms

roughness of the film surface was 6.78 nm and 14.3 nm for 1×1 μm and 5×5 μm area, respectively (Fig S2).

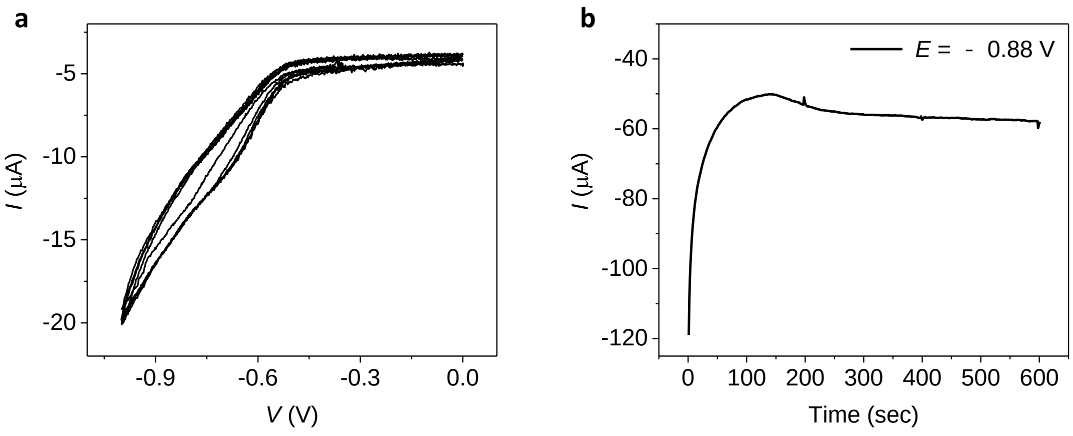

Supplementary Figure 1. Electrochemical deposition of Cr-PBA in a aqueous solution a Cyclic voltammetry curve for the observation of reductive reaction from 0.0 V to −1.0 V **b** The generated current recorded for 10 min during the deposition of the Cr-PBA film at $E = -0.88$ V.

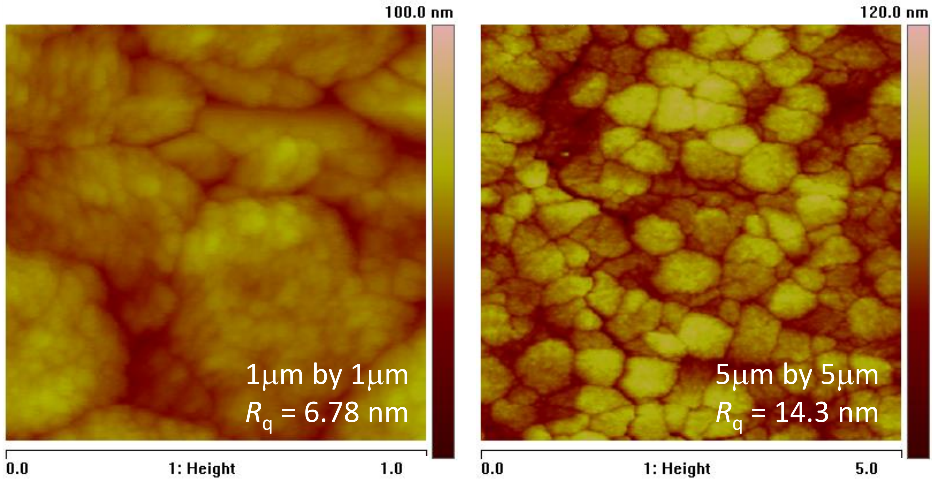

Supplementary Figure 2. AFM images of the Cr-PBA film surface. The estimated roughness of the Cr-PBA film is 6.78 nm and 14.3 nm for 1×1 μm and 5×5 μm area, respectively.

Magnetic properties of Cr-PBA

The magnetic characteristics of the ECD deposited Cr-PBA film were studied by using SQUID-VSM (Quantum Design) immediately after the deposition. Magnetic hysteresis of the Cr-PBA film measured for in-plane applied magnetic field at 100 K exhibits a coercivity of about 2.5 mT (Fig. 1f). The gradual flip of the magnetization shows polycrystalline characteristic of the Cr-PBA film. In order to examine the stability of sample, we measured magnetic hysteresis of Cr-PBA after keeping the sample for one month in an ambient air. We didn't observe any discernible change in magnetization. To determine the critical temperature of our Cr-PBA film by using Arrott plot method, we plotted M^2 vs B/M for various temperatures from 190 K to 250 K as shown in Fig. S3. The plot should display a straight line passing through the origin at the transition temperature. The obtained transition temperature of our Cr-PBA film was about 230 K as shown in the inset of Fig. S3. This value is close to $T_c \sim 240$ K of the crystalline powder samples [1]. This discrepancy is likely due to a slight change in the stoichiometric ratio between Cr^{2+} (S = 2) and Cr^{3+} (S = 2/3), as it changes net spin numbers, variations of crystal field, and the strength of superexchange coupling [2]. The temperature-dependent saturation magnetization curves exhibit typical behavior of temperature dependent magnon excitation. Fig. S4a displays a plot for the Bloch $T^{3/2}$ law exhibiting a good linearity over the wide range of temperature. Fitting with $M(T) = M_0(1-aT^{3/2})$ provides the value of slope ~ 2.6×10^{-4} $K^{-3/2}$. This value of slope is higher than those of Ni (7.5×10^{-6} $K^{-3/2}$) [3] and YIG (5.8×10^{-5} $K^{-3/2}$). This might suggest that the excitations of magnons with low wave vectors are more effective in our Cr-PBA film. Fig. S4b exhibits temperature-dependent magnetization measured with applying various magnetic field from 0.001 T to 0.5 T. The slope of Block spin wave decreases with decreasing magnetic field. This could be due to the fact that

smaller size of domain formed at low magnetic field would not be adequate for the effective excitation of low-energy and long-wavelength spin waves.

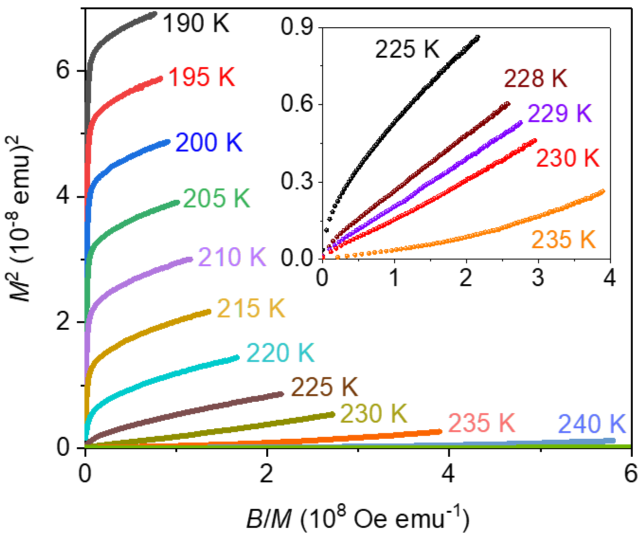

Supplementary Figure 3. Arrott plots for the ECD deposited Cr-PBA film. The plots of M^2 vs B/M for various temperatures from 190 K to 250 K with 5 K interval. The inset shows the Arrott plots at several temperatures in the vicinity of the T_c.

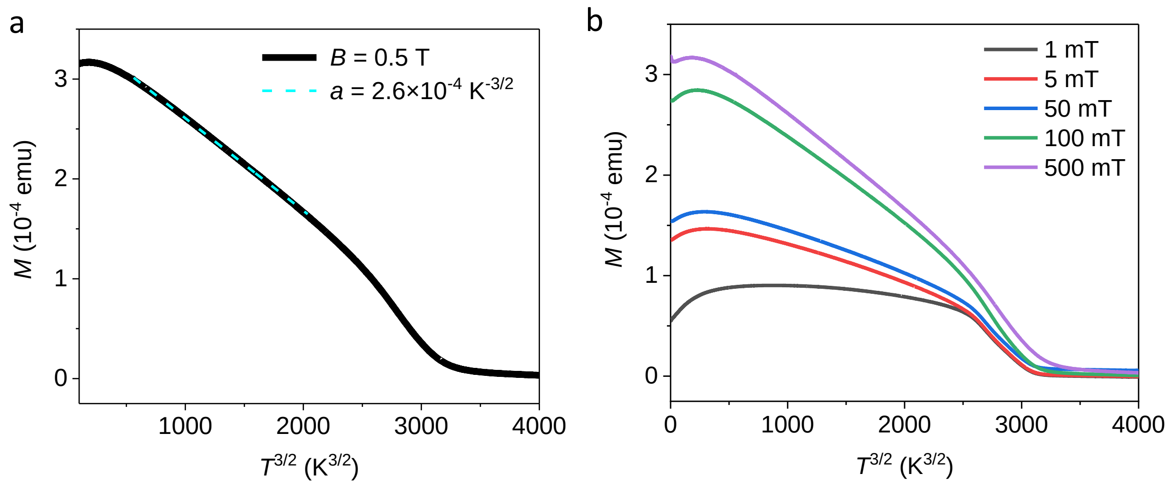

Supplementary Figure 4. Plot of the Block $T^{3/2}$ law. a Temperature dependent saturation magnetization as a function of $T^{3/2}$ displaying a good linearity over the wide range of temperature. Measurement was done with applying magnetic field of $B = 0.5$ T. Fitting with $M(T) = M_0(1-aT^{3/2})$ provides the value of slope ~ 2.6×10^{-4} $K^{-3/2}$, which is much higher than those of inorganic magnets.

b Temperature dependent magnetization with applying various magnetic field from 0.001 T to 0.5 T.

Supplementary Note2. Temperature calibration and SSE characterization

Thermal conductivity of Cr-PBA

To determine the thermal conductivity of the Cr-PBA film, we adopted the differential 3ω method [4,5]. The differential 3ω technique especially has been used to determine the anisotropic thermal conductivity of thin films because it allows measurement of the temperature drop across only the intended film with removing uncertainties in the thermal properties of the other layers [4,5]. As shown in Fig. S5a, we deposited a Cr-PBA film (4 μm) on the half of the Cr (10 nm) /*p*-Si substrate. Then, the thin insulating layer of parylene C was coated by using a parylene coater (Alpha plus). Thickness of the parylene layer was about 400 nm, guaranteeing electrical isolation of the gold heater to function as a thermometer. In this way, the sample (Parylene/Cr-PBA/Cr/*p*-Si) and the reference (Parylene/Cr/*p*-Si) were fabricated on the same substrate. Then, 100 μm × 5 mm gold heater lines were patterned on top of both sides of the sample and reference sample parts. Measurements of both the third harmonic voltage ($V_{3\omega}$) and the first harmonic voltage ($V_{1\omega}$) enable to determine the cross-sectional temperature drop (ΔT) from the relation,

$$\Delta T = 2R\frac{dT}{dR}\frac{V_{3\omega}}{V_{1\omega}} \qquad \text{eq(1)}$$

where R is the resistance of a Au heater line. Fig. S6b displays estimated cross-sectional temperature drop in the sample and reference, respectively at 100 K. The difference of temperature drop between sample and reference indicates an exclusive temperature drop across the Cr-PBA film (ΔT_{film}). For the heater power (P) of 88 mW, the estimated ΔT_{film} is ~ 0.32±0.01 K. Then, the thermal conductivity of the film can be determined by the following equation,

$$\kappa = P \cdot d_{\text{f}} / (w_{\text{h}} l_{\text{h}} \cdot \Delta T_{\text{film}}) \qquad \text{eq(2)}$$

where d_f, w_h, and l_h are the thickness of the film, the width and length of the Au heater, respectively. The thermal conductivity of the Cr-PBA film was estimated to be 2.18±0.01 W/mK, when the applied heating power was 88 mW. We repeated measurements with increasing the applied heater power as shown in Fig. S5c. The obtained average value of the thermal conductivity was 2.17±0.01 W/mK.

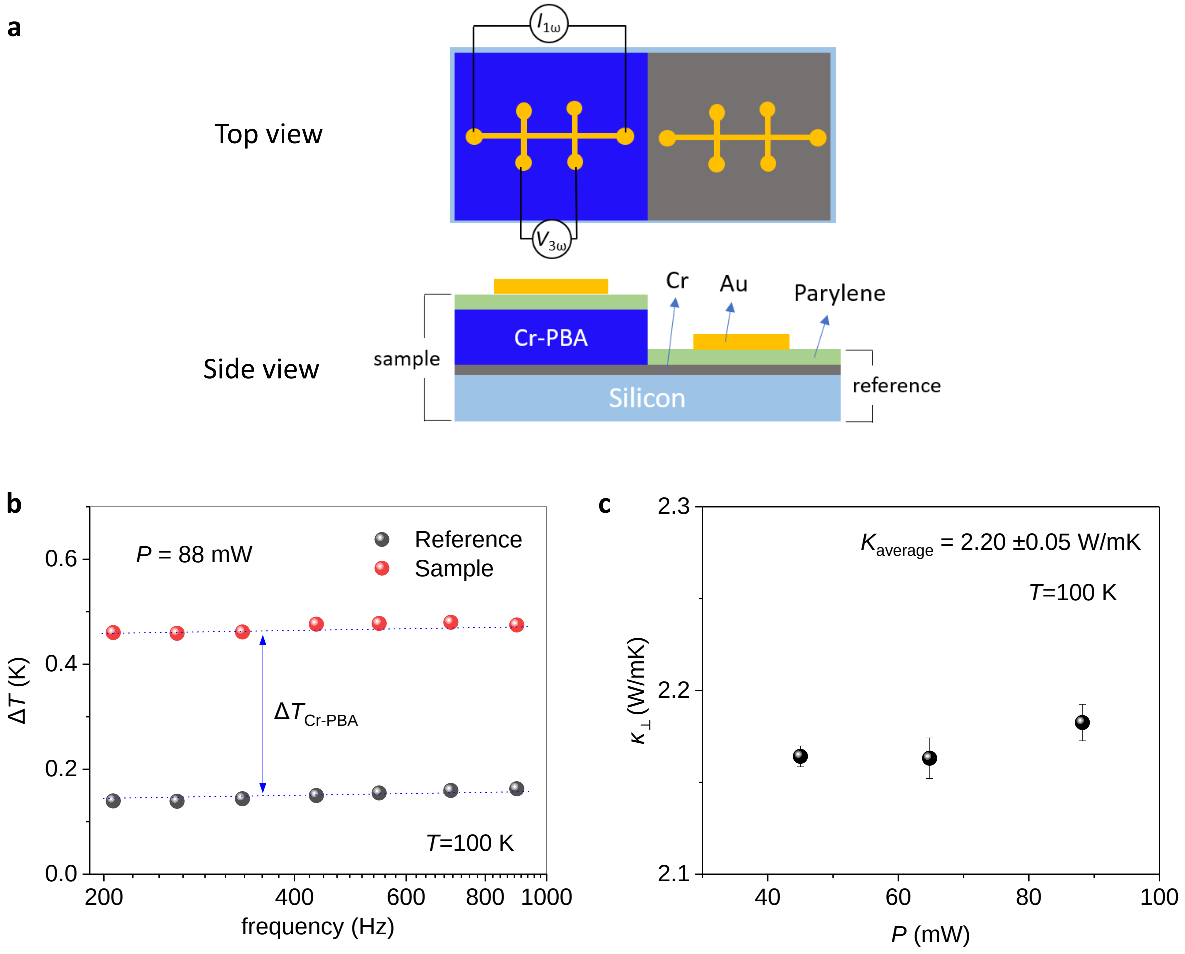

Supplementary Figure 5. Differential 3ω measurements for the determination of the thermal conductivity of the Cr-PBA film. a Schematic illustration of top and cross-section view for the sample and the reference which are fabricated on the same substrate. **b** 2ω oscillation (ΔT) as a function of logarithm frequency. Both the sample and reference display nearly the same linearity.

The difference of the temperature (ΔT_{film}) is calculated to be 0.32 K. **c** Estimated the thermal conductivity as increasing the heater power. The error bars indicate the standard deviations of κ estimated from the standard deviations of the measured ΔT_{film} between the sample and reference.

Temperature calibration

To estimate the spin Seebeck coefficient (S_{LSSE}) of our STE devices, a temperature gradient applied on the Cr-PBA film need to be obtained. We employed the top Au heating layer as an in-situ temperature sensor[6,7]. Thus, heat loss at the interface between a STE device and a temperature sensor can be ignored allowing a precise calibration of temperature. The measurements were conducted in a PPMS chamber under the precise control of the system-temperature. Bottom of the substrate of the STE device was attached to the gold plate of the PPMS sample puck with a thermal M grease. The temperature of the top Au layer was estimated based on the temperature dependent resistance of the Au layer. Fig. S6a displays temperature dependent resistance of the Au layer. The slope of the R-T curve is 0.363 (Ω/K). The measurement was done with source current, 0.1 mA, to exclude Joule heating. Fig. S6b displays the time dependent resistance of the Au layer during the heating with stepwise currents. As the heating current was increased in step, the resistance of the Au layer was also increased stepwise. The resistance of the Au line was immediately stabilized without suffering an oscillation because the heating power of the micro-scale Au wire is not high enough to affect the system temperature of the PPMS. For the applied current of 10 mA, a temperature stabilization takes less than min. The temperature of the top Au heater for the heating current of 15 mA is estimated to be 104.91 K.

We then calculated the temperature gradient applied in each layer by using the Fourier's Law,

$$q_x = -\kappa A \frac{dT}{dx} \qquad \text{eq(3)}$$

where q_x, κ, A are the heat flux (W), thermal conductivity (W/mK), and surface area, respectively. Thus, the temperature difference applied on each layer will be proportional to d/κ, where d is the thickness of each layer. Here, we ignored the interfacial temperature drop as the interfaces are atomically contacted. The estimation of temperature gradient on Cr-PBA may include slight uncertainty as the relative thickness of Cr-PBA is very thin compared to the interval where we measured temperature[8]. The reference values of κ are used for the thermal conductivity of each layer except the Cr-PBA layer. The unknown thermal conductivity of Cr-PBA was obtained based-on the differential 3ω method. The calculated temperature difference on each layer for various heating currents is summarized in Table 1. Fig. S7 illustrates the temperature gradient applied in each layer of our STE device for a heating current of 15 mA.

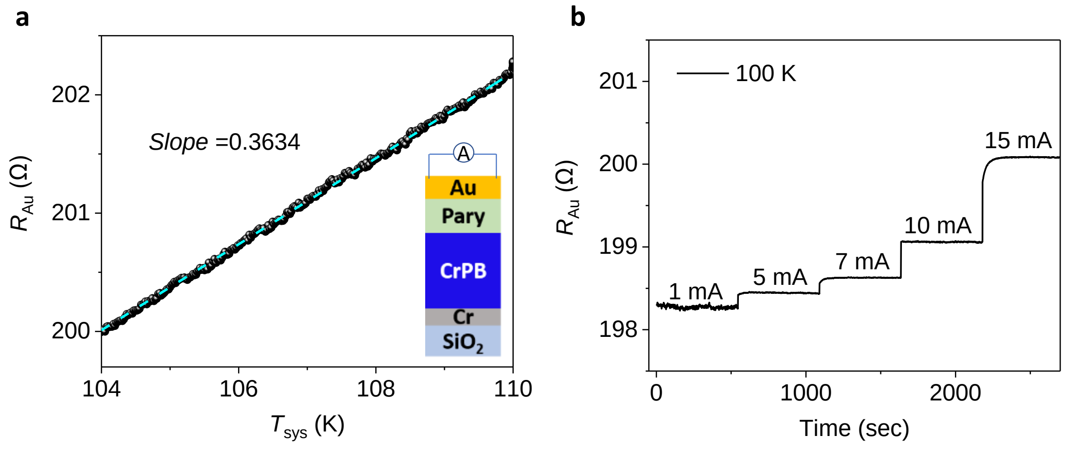

Supplementary Figure 6. Temperature calibration using the on-chip Au heater line. a The temperature dependent resistance of the Au heater recorded for the calibration of the temperature at the top Au layer. **b** The resistance of the Au line recorded during the stepwise increase of a Joule heating current at 100 K.

	κ (W/mK)	Thickness	$\triangle T_{10mA}$	$\triangle T_{15mA}$	$\triangle T_{20mA}$
T_{Au}-T_{system}		20 nm	2.16 K	4.91 K	10.18 K
Al_2O_3	30	130 nm	0.00098 K	0.0022 K	0.0046 K
Parylene	0.082	400 nm	1.1 K	2.5 K	5.2 K
**Cr-PBA**	2.2	1.4 μm	**0.15 K**	**0.34 K**	**0.72 K**
Cr	93.9	10 nm	0.000024 K	0.000055 K	0.00011 K
SiO_2	1.4	300 nm	0.049 K	0.11 K	0.23 K
Si	150	500 μm	0.76 K	1.72 K	3.57 K

Supplementary Table 1. The calculated temperature difference applied in each layer. The temperature difference applied in each layer can be calculated by the heat flux relation between thickness and thermal conductivity ($\Delta T \sim d/\kappa$).

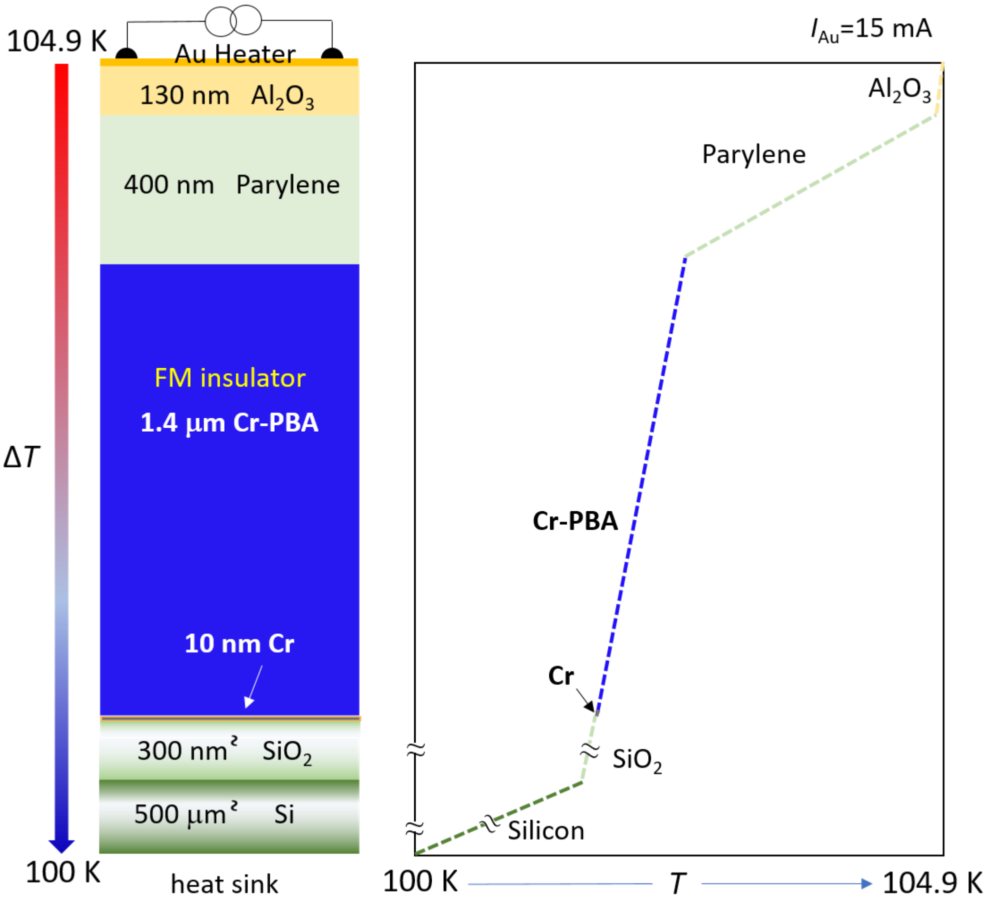

Supplementary Figure 7. Schematic illustration of the temperature gradient in the STE device. The schematic figure of the temperature gradient in each layer of the STE device. The smaller the thermal conductivity is, the higher temperature gradient occurs. The studied STE device has the highest temperature gradient on the parylene layer, followed by the Cr-PBA film.

Characterization of longitudinal spin Seebeck effect (LSSE) coefficient

The obtained LSSE signal for different heating currents (I_{heater}) in the on-chip Au heater can provide precise estimation of the LSSE coefficient. Because the heat flux is generated by Joule heating, the resistance of the Au heater (R_{Au}), the temperature difference applied in Cr-PBA ($\Delta T_{Cr\text{-}PBA}$) and the obtained V_{LSSE} are all proportional to $\sim I^2$ as shown in Fig. S8. Then, the spin Seebeck coefficient, defined as $S_{LSSE} = E_{ISHE}/\nabla T = (\Delta V_{LSSE}/L)/(\Delta T_{Cr\text{-}PBA}/d)$ (where L is the interval between voltage probes and d is the thickness of the Cr-PBA film), can be estimated from the slope of $\Delta V_{LSSE}/\Delta T_{Cr\text{-}PBA}$. The obtained spin Seebeck coefficient of the Cr-PBA/Cr device at 100 K was S_{LSSE} = 18.2 nV/K for 1.4 μm thick Cr-PBA. The spin Seebeck coefficient was also estimated with varying thickness of Cr-PBA, which exhibits general tendency of increment with increasing thickness of Cr-PBA (Fig. S9). The observed ΔV_{LSSE} can be also induced from other effects associated with heating. Anomalous Nernst effect (ANE) has been considered as one of the main sources to contribute V_{LSSE}, because proximity induced magnetism at the interface could induce heat gradient induced voltage with the same spatial symmetry with V_{LSSE}. Thus, we measured anomalous Hall effect (AHE) to investigate a proximity effect in our heterojunction (Fig. S10). The hall bar structure was patterned to measure Hall voltage (V_{xy}). We applied 1 μA of source currents to exclude Joule heating during the Hall measurements. Result show the absence of a proximity effect in the Cr layer (Fig. S10). Thus, the observed V_{LSSE} in our device is not associated with ANE and mainly originates from LSSE.

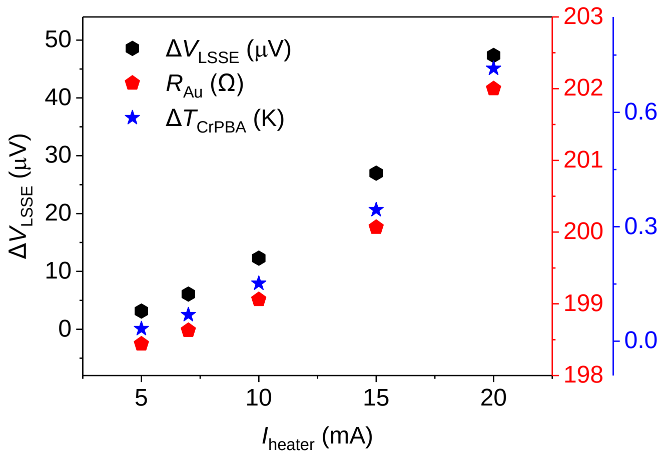

Supplementary Figure 8. Heating power dependence. ΔV_{LSSE} (black circles), resistance of the Au layer (R_{Au}, red pentagons), and the temperature difference in Cr-PBA ($\Delta T_{\mathrm{Cr\text{-}PBA}}$, blue stars) measured with increasing the heating current at 100 K.

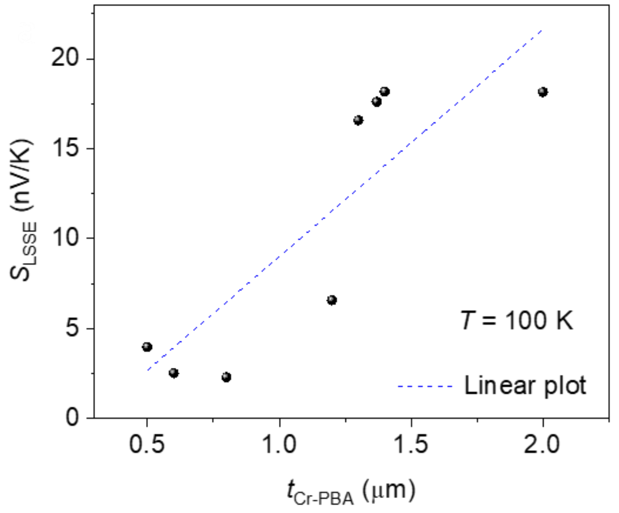

Supplementary Figure 9. **Thickness dependence of spin Seebeck coefficient.** The spin Seebeck coefficient measured for the Cr-PBA($t_{\mathrm{Cr\text{-}PBA}}$)/Cr(10 nm) devices with various thicknesses of Cr-PBA. The error bar, standard deviation of the estimated S_{LSSE}, in each device was smaller than the size of symbol in the plot.

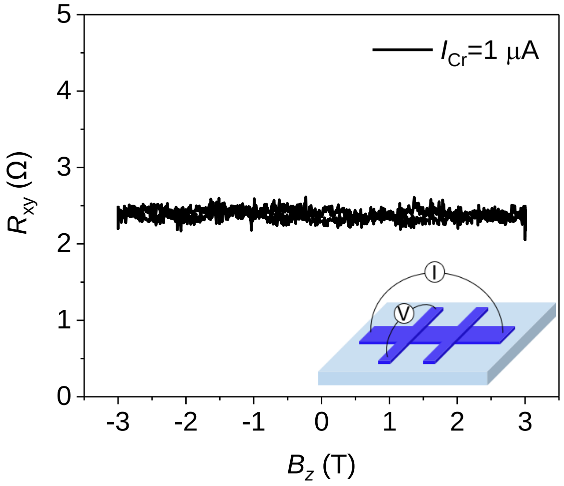

Supplementary Figure 10. Anomalous Hall effect in the Cr-PBA/Cr heterojunction. The observed V_{LSSE} is often involved with an experimental artifact from the anomalous Hall effect (AHE) because the proximity effect could induce spontaneous magnetization in Cr layer. The AHE was measured with standard Hall geometry of the Cr-PBA/Cr bilayer at 100 K. Results clearly display absence of AHE.

Temperature- and field-dependent LSSE characteristics for different thickness of Cr-PBA

We investigated the characteristics of LSSE as functions of temperature and magnetic field with varying thickness of Cr-PBA film. Fig. S11a shows the temperature dependent LSSE with three different thicknesses of Cr-PBA films measured by sweeping magnetic field between $B_y = -0.5$ T and 0.5 T at each temperature. The peak of temperature dependent V_{LSSE} appears around 50 K for 2 μm thick Cr-PBA and gradually shifts to higher temperature as decreasing thickness of Cr-PBA film. The peak is almost suppressed for 0.5 μm thick film. This behavior is similar to what have

been observed in previous works with YIG film[9]. To investigate contribution of phonon on the spin Seebeck effect, we measured the temperature dependence of the thermal conductivity of Cr-PBA. Results show that the thermal conductivity of the Cr-PBA film increases with increasing temperature within the measurement window (up to room temperature) as shown in Fig. 11b. Thus, we can confirm that the observed peak of $V_{\mathrm{LSSE}}(T)$ is a size effect associated with magnon propagation length. Fig. S12a-c shows magnetic field dependent $V_x(B_y)$ measured at $T = 100$ K and 300 K, while applying vertical temperature gradient (∇T_z). The linear suppression of the measured voltage was also observed even above the Curie temperature of Cr-PBA, which can be attributed to the ordinary Nernst effect in Cr layer because it has the same symmetry of V_{LSSE}. Assuming the Nernst effect is nearly temperature independent for metal, we subtracted the linear slop of Nernst effect measured at 300 K for each sample to the measured $V_x(B_y)$ in order to obtain the field-dependent LSSE signal separately. Fig. S12d displays the extracted V_{LSSE} as a function of applied magnetic field for different thickness of Cr-PBA. Results display high-field suppression ratio of the LSSE signal tends to increase with increasing thickness of Cr-PBA film, as observed in previous works[10,11]. However, precise subtraction of the Nernst effect in our devices is unattainable task because the applied ΔT in the thin Cr layer will be largely temperature dependent. We estimated ΔT in each layer by using the Fourier's law. But, thermal conductivities in every layer are generally temperature dependent as in Cr-PBA, whose κ value at 300 K is nearly twice of the value at 100 K (Fig. S11b). Thus, the applied ΔT in the thin Cr layer is temperature dependent, so does the size of Nernst voltage. Therefore, actual high-field suppression in V_{LSSE} could be considerably different from the estimated $V_{\mathrm{LSSE}}(B_y)$ shown in Fig. S12d. Fig. S13 shows field dependent LSSE signal measured at various temperatures for the device with 1.4 μm thick Cr-PBA, after subtracting the linear fit to ordinary Nernst effect measured at 300 K. Results exhibit

no consistent tendency of the field-dependent suppression ratio as varying system temperature. We also tested magneto-resistance (MR) of the Au and Cr layer to confirm parasite effect that may contribute to the observed V_{LSSE}, especially in high magnetic field (Fig. S14). For the measurement of MR in the Cr layer, we applied from 1 μA to 100 μA to exclude Joule heating and applied the external magnetic field up to 7 T. The observed resistance change at high magnetic field was less than 0.043 %. For the Au heater line, we measured MR by applying 10 mA to examine heating variation as increasing magnetic field. The change of resistance was less than 0.037 %, which does not contribute to the relatively larger variation in V_{LSSE} at high magnetic field.

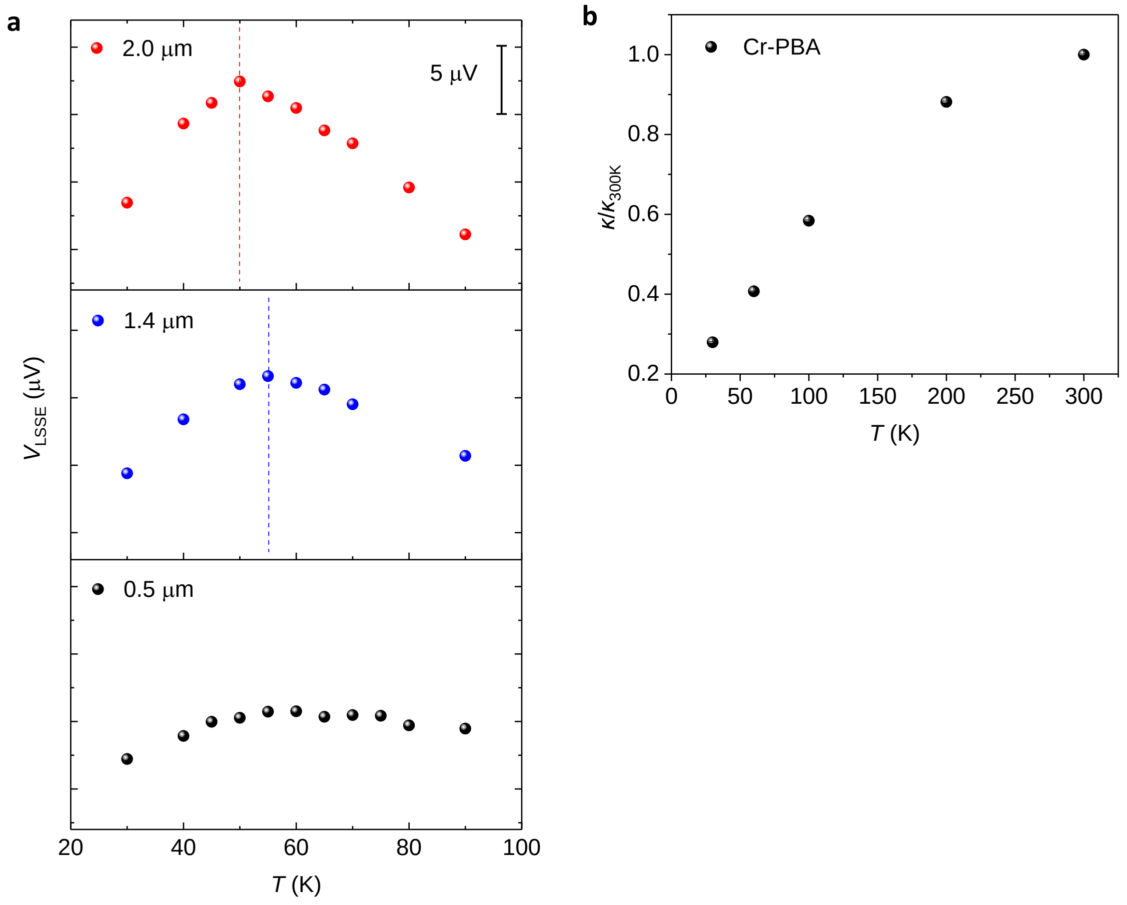

Supplementary Figure 11. Temperature- and field-dependent characteristics of LSSE. a The temperature dependence of LSSE in the three different thicknesses of $t_{Cr\text{-}PBA}$ = 2.0, 1.4, 0.5 μm,

measured by sweeping between $B = \pm 0.5$ T. **b** The temperature dependence of the thermal conductivity of Cr-PBA. The error bars for the estimated κ were smaller than the size of symbol in the plot.

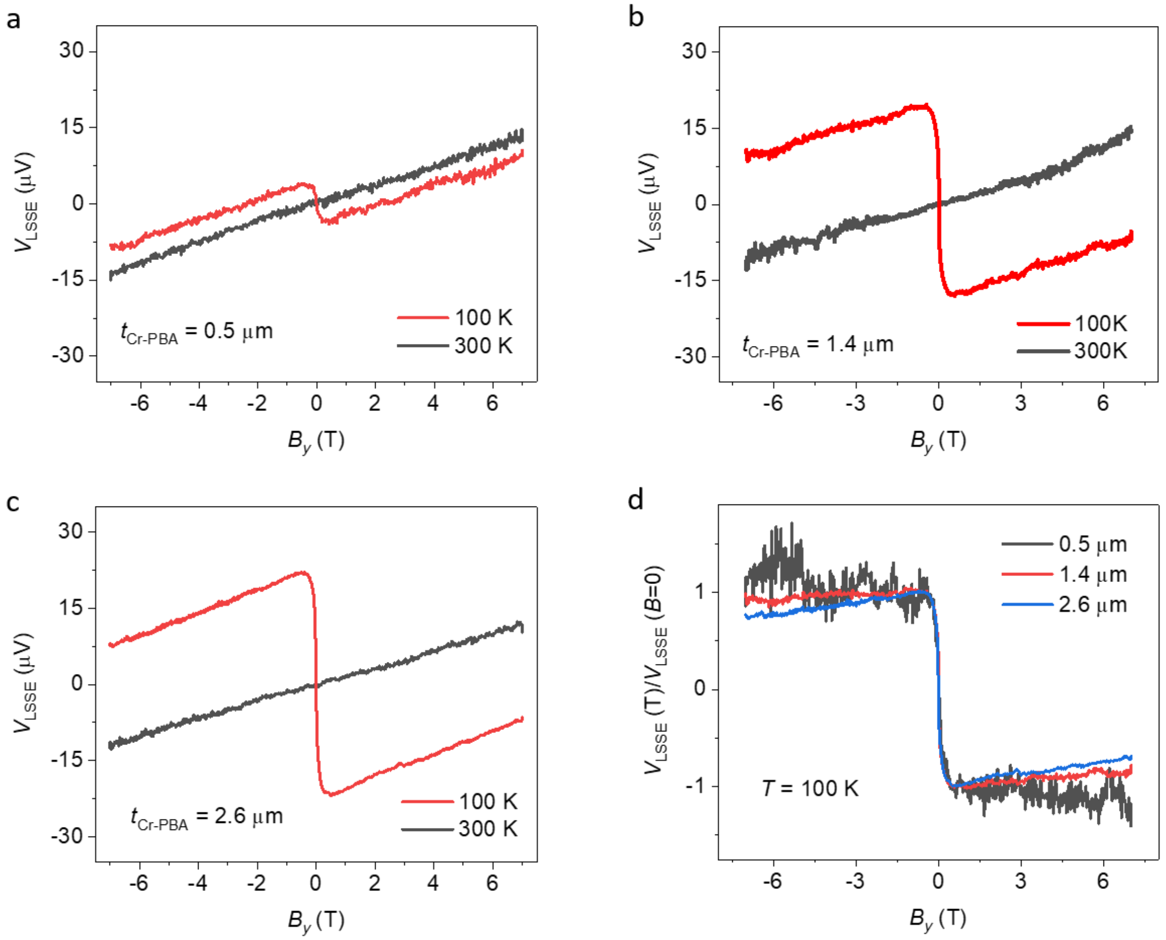

Supplementary Figure 12. Nernst effect of Cr layer and field-dependent LSSE. a-c LSSE signal along with Nernst effect (at 100 K) and purely Nernst effect (at 300 K) measured for the Cr-PBA/Cr heterojunctions with different thicknesses of 0.5 μm(**a**), 1.4 μm(**b**), and 2.6 μm(**c**). **d** The magnetic field dependent LSSE signals measured for different thicknesses of Cr-PBA films at 100 K. The linear fits to the Nernst effects measured for each sample at 300 K were subtracted.

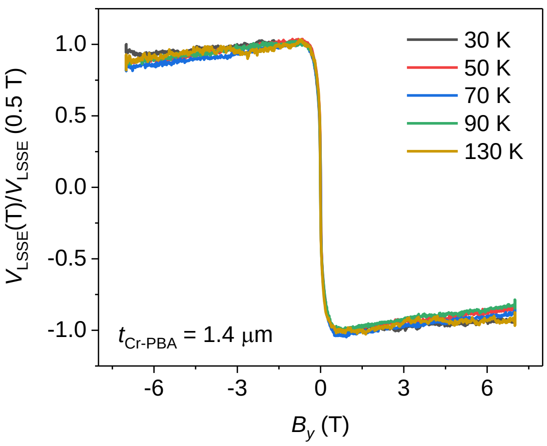

Supplementary Figure 13. Temperature dependence of high-field suppression of LSSE. High-field suppression of V_{LSSE} at various temperatures measured for the device with 1.4 μm thick Cr-PBA.

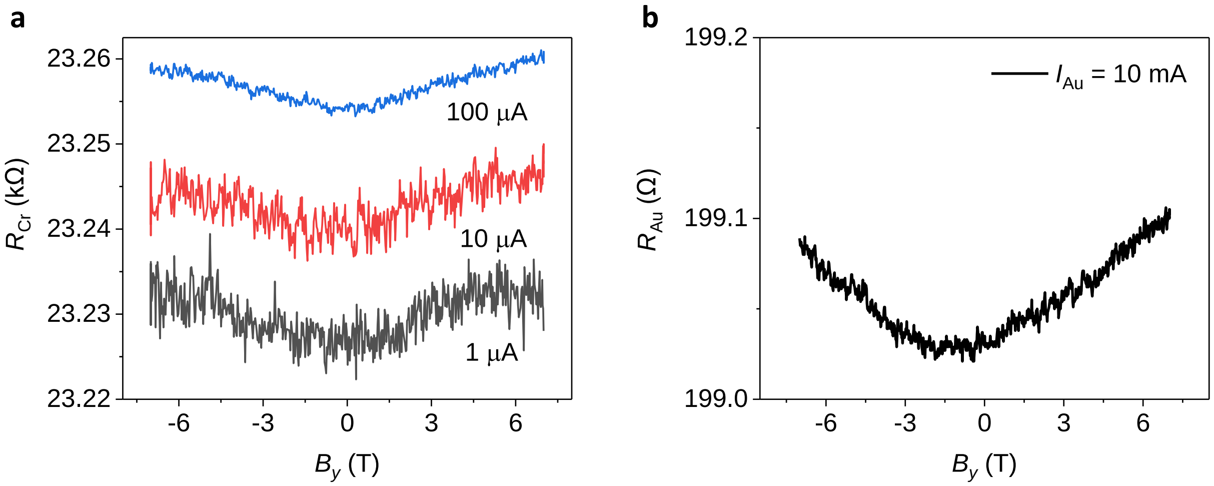

Supplementary Figure 14. MRs under the applied field *B*$_y$. The MRs of the Cr layer (**a**) and the top Au heater line (**b**) measured for the applied in-plane magnetic field (B_y). Results display negligible MRs in both Cr and Au electrodes. Thus, MRs of the electrodes have negligible effect in the calibration of the measured V_{LSSE}.

Supplementary Note3. FMR and FMR-ISHE measurements

We studied the generation, propagation, and detection of magnons in the Cr-PBA/Cr heterojunction further through ferromagnetic resonance (FMR) and FMR with inverse spin Hall effect (FMR-ISHE) experiments. Both FMR and FMR-ISHE measurements were done with coplanar waveguide. The FMR spectra were recorded with sweeping the external magnetic field at microwave frequencies in the range of 3-19 GHz (Fig. 4a). The resonance field (B_R) and linewidth (ΔB) of FMR spectra were extracted from the first derivative of Lorentzian, as follows

$$\frac{dP}{dB_{DC}} = K_1 \frac{4\Delta B(B-B_R)}{[4(B-B_R)^2+(\Delta B)^2]^2} - K_2 \frac{[(\Delta B)^2-4(B-B_R)^2]}{[4(B-B_R)^2+(\Delta B)^2]} + \text{slope}B + \text{offset} \qquad \text{eq(4)}$$

The dependence of the resonance field (B_R) on the microwave frequency can be well described with the Kittel equation, $\nu = \frac{\gamma}{2\pi}[B_R(B_R + \mu_0 M_s)]^{1/2}$, where ν is microwave frequency, γ is the gyromagnetic factor, B_R is a resonance field, M_s is a saturation magnetization. Fitting with Kittel equation produces M_s = 12.676 kA/m and g-factor = 1.96 (Fig. 4b). The frequency dependence of ΔB of FMR spectra in Cr-PBA/Cr heterostructures can provide the effective Gilbert damping constant (α_{eff}) following the relation,

$$\Delta B = \Delta B_0 + \frac{4\pi\nu\alpha_{\text{eff}}}{\sqrt{3}\gamma} \qquad \text{eq(5)}$$

where, ΔB_0 denotes an inhomogeneous broadening by structural imperfections. The obtained effective Gilbert damping constant for the Cr-PBA (1.4 μm)/Cr (10 nm) heterojunction was $\alpha_{\text{eff}} = 7.5 \times 10^{-4}$ (Fig. 4c). We also recorded FMR spectra of Cr-PBA/Cr heterojunctions with varying thickness *d* of Cr-PBA films. The frequency dependence of FMR spectra provides α_{eff} for Cr-PBA/Cr heterojunctions of different *d*. Then, intrinsic damping constant (α_0) of the Cr-PBA film

and spin mixing conductance ($g_{\text{eff}}^{\uparrow\downarrow}$) of the heterojunction can be estimated following the equation [12],

$$\alpha_{\text{eff}} = \alpha_0 + \Delta\alpha = \alpha_0 + \frac{g\mu_B}{4\pi M_s d} g_{\text{eff}}^{\uparrow\downarrow}. \quad \text{eq (6)}$$

where $\Delta\alpha$ is the additional Gilbert damping caused by spin pumping. Fig. S11 shows thickness dependence of the measured effective damping constant (α_{eff}). Here, we assume constant value of the spin mixing conductance, $g_{\text{eff}}^{\uparrow\downarrow}$, because the fabrications of Cr-PBA/Cr heterostructures were done under the same condition. Fitting with the equation 6 produces estimated values of α_0 = $(2.4\pm0.67)\times10^{-4}$ and $g_{\text{eff}}^{\uparrow\downarrow} = (6.5\pm0.52)\times10^{18}$ m^{-2}, which are comparable to those of the YIG and YIG/Pt [12,13].

The conversion of spin angular momentum into an electric current in the Cr-PBA/Cr heterojunction was studied further through FMR-driven ISHE as illustrated in the Fig. 4d. Fig. 4e displays measured V_{ISHE} at continuous microwave f = 9 GHz in the Cr-PBA/Cr heterojunction. Both ΔB and B_R of V_{ISHE} are in consistent with those of FMR spectra shown in Fig. 4a. This clearly suggests that the measured V_{ISHE} originates from FMR-generated magnon flow in the Cr-PBA film. The transferred spin current density j_s^0 at the interface can be determined from the relation,

$$J_s^0 = \frac{g_{\text{eff}}^{\uparrow\downarrow}\gamma^2 B_1^2 \hbar\left[4\pi M_s\gamma\sin\theta_M^2 + \sqrt{(4\pi M_s)^2\gamma^2 + 4\omega^2}\right]}{8\pi\alpha_{\text{eff}}^2\left[(4\pi M_s)^2\gamma^2\sin\theta_M^4 + 4\omega^2\right]} \quad \text{eq(7)}$$

where B_1 is rf field and θ_M is the angle between the magnetization direction and the film normal. Then, the spin Hall angle ($\theta_{\text{SHE}}^{\text{Cr}}$), a figure of merit for the conversion between the transferred spin current and charge current, can be estimated following the relation,

$$V_{\mathrm{ISHE}} = \frac{2e}{\hbar}\frac{\theta_{\mathrm{SHE}}^{\mathrm{Cr}}\lambda_{\mathrm{s}}^{\mathrm{Cr}}\omega J_{\mathrm{s}}^{0}}{\sigma^{\mathrm{Cr}}d^{\mathrm{Cr}}}\tanh\left(\frac{2d^{\mathrm{Cr}}}{\lambda_{\mathrm{s}}^{\mathrm{Cr}}}\right) \quad \text{eq(8)}$$

where $\lambda_{\mathrm{s}}^{\mathrm{Cr}}$ and σ^{Cr} are the spin diffusion length and conductivity of the Cr layer, respectively. We took $\lambda_{\mathrm{s}}^{\mathrm{Cr}} = 2.1$ nm from literature [14] and the value of σ^{Cr} from the 4-terminal measurement. The obtained spin Hall angle ($\theta_{\mathrm{SHE}}^{\mathrm{Cr}}$) ~ -0.014. This value is within the range of $\theta_{\mathrm{SHE}}^{\mathrm{Cr}}$ values in the literature [14,15]. In short, the studies of FMR and FMR-ISHE demonstrate the effective generation of spin flow in Cr-PBA and spin-pumping process into the adjacent Cr layer.

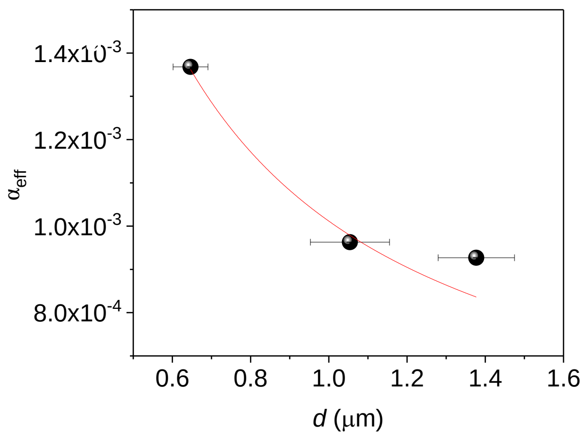

Supplementary Figure 15. Thickness dependent damping constants of Cr-PBA/Cr heterojunctions. For the calculation of the intrinsic damping constant of the Cr-PBA film, we measured FMR for Cr-PBA thickness ranging from 650 nm to 1.4 μm.

References

1 Mallah, T., Thiebaut, S., Verdaguer, M. & Veillet, P. High-T_c Molecular-Based Magnets: Ferrimagnetic Mixed-Valence Chromium(III)-Chromium(II) Cyanides with T_c at 240 and 190 Kelvin. *Science* **262**, 1554-1557, (1993).

2 Ruiz, E., Rodríguez-Fortea, A., Alvarez, S. & Verdaguer, M. Is it possible to get high T_C magnets with Prussian blue analogues? A theoretical prospect. *Chem. Eur. J.* **11**, 2135-2144, (2005).

3 Chen, C.-W. *Magnetism and Metallurgy of Soft Magnetic Materials*. p51 (Dover, New York, 1977).

4 Cahill, D. G. Thermal conductivity measurement from 30 to 750 K: the 3ω method. *Rev. Sci. Instrum.* **61**, 802-808, (1990).

5 Yang, B., Liu, J. L., Wang, K. L. & Chen, G. Simultaneous measurements of Seebeck coefficient and thermal conductivity across superlattice. *Appl. Phys. Lett.* **80**, 1758-1760, (2002).

6 Wu, S. M., Fradin, F. Y., Hoffman, J., Hoffmann, A. & Bhattacharya, A. Spin Seebeck devices using local on-chip heating. *J. Appl. Phys.* **117**, 17C509, (2015).

7 Oh, I. *et al.* Solution-Processed Ferrimagnetic Insulator Thin Film for the Microelectronic Spin Seebeck Energy Conversion. *ACS Appl. Mater. Interfaces* **10**, 28608-28614, (2018).

8 Uchida, K. *et al.* Longitudinal spin Seebeck effect: from fundamentals to applications *J Phys.: Condens. Matter* **26**, 343202, (2014).

9 Guo, E. J. *et al.* Influence of Thickness and Interface on the Low-Temperature Enhancement of the Spin Seebeck Effect in YIG Films. *Phys. Rev. X* **6**, 031012, (2016).

10 Ritzmann, U. *et al.* Magnetic field control of the spin Seebeck effect. *Phys. Rev. B* **92**, 174411, (2015).

11 Kikkawa, T. *et al.* Critical suppression of spin Seebeck effect by magnetic fields. *Phys. Rev. B* **92**, 064413, (2015).

12 Jungfleisch, M. B. *et al.* Thickness and power dependence of the spin-pumping effect in $Y_3Fe_5O_{12}$/Pt heterostructures measured by the inverse spin Hall effect. *Phys. Rev. B* **91**, 134407, (2015).

13 Collet, M. *et al.* Generation of coherent spin-wave modes in yttrium iron garnet microdiscs by spin-orbit torque. *Nat. Commun.* **7**, 10377, (2016).

14 Qu, D., Huang, S. Y. & Chien, C. L. Inverse spin Hall effect in Cr: Independence of antiferromagnetic ordering. *Phys. Rev. B* **92**, 020418, (2015).

15 Du, C. H., Wang, H. L., Yang, F. Y. & Hammel, P. C. Systematic variation of spin-orbit coupling with *d*-orbital filling: Large inverse spin Hall effect in 3*d* transition metals. *Phys. Rev. B* **90**, 140407(R), (2014).
